# Longitudinal study of the ecap measured in children with cochlear implants

**DOI:** 10.1016/S1808-8694(15)30837-5

**Published:** 2015-10-18

**Authors:** Liege Franzini Tanamati, Maria Cecília Bevilacqua, Orozimbo Alves Costa

**Affiliations:** 1 Master’s degree, speech therapist.; 2 Full professor, speech therapist, Speech Therapy Course.; 3 Full professor, professor in the Speech Therapy Course. Faculdade de Medicina da Universidade de São Paulo.

**Keywords:** child, cochlear implant, cochlear nerve

## Abstract

In children with cochlear implant (CI), the recording of the electrically evoked compound action potential (ECAP) of the auditory nerve represents an option to assess changes in auditory nerve responses and the interaction between the electrode bundle and the neural tissue over time. **Aim:** To study ECAP in children during the first year of CI use. **Materials and methods:** The ECAP characteristics have been analyzed in 13 children implanted younger than three years of age. Series study. **Results:** During the first year of CI use there was a significant statistical raise in the N1 peak amplitude, in basal electrodes, between the second and third return visits. There were not any significant differences obtained for N1 peak, latency, slope, p-NRT or recovery time, in the return visits. **Conclusion:** During the first year of CI use, the electrical stimulation provided by the intracochlear electrodes did not cause significant changes to ECAP characteristics, except for an increase in N1 peak amplitude.

## INTRODUCTION

There is considerable variability in the auditory performance of cochlear implant (CI) users. This has been attributed to the features of the central and peripheral auditory system that result from the impact of sensorineural hearing loss on afferent neural structures.^1^, ^2^

Auditory nerve ganglion cells are considered the elements that effectively respond to electrical stimuli emitted by cochlear implants. Thus, the number, distribution, and functional status of these cells define an individual’s ability to benefit from a cochlear implant.^3^

Post-mortem histopathological studies provide information about the number and distribution of ganglion cells in cochlear implant users. These studies, however, are unable to assess the functional status and the neurophysiological properties of these neural elements when stimulated by cochlear implant electrodes.

Recording the electrically evoked compound action potential (ECAP) is a direct method for assessing in vivo the functional status of ganglion cells and other auditory neural structures. Currently available cochlear implant models record and analyze the ECAP by using a bidirectional communication system between the internal and external component, which can stimulate and pick up a response from auditory nerve fibers. The software that performs these recordings is the Neural Response Telemetry (NRT), for cochlear implants of the brand Nucleus (Cochlear Corporation).

ECAP features, measured in different intracochlear electrodes, express the permeability of neural elements to respond to an electrical stimulus. For clinical research, assessing these features longitudinally helps monitor the changes in interface electrodes and neural tissues across the time of use of a cochlear implant.

Longitudinal research in animals and humans have described the first year of cochlear implant use as a period in which the main changes in the auditory system occur as a response to device-generated electrical stimuli.^4^

Longitudinal studies of cats with implants revealed changes in electrically evoked auditory brainstem response (EABR) wave amplitude and thresholds and the ECAP, and also changes in the duration of the refractory period of the auditory nerve.^5^, ^6^, ^7^

Studies in humans have shown different results in the stabilization of ECAP features, especially its amplitude, extrapolated threshold (p-NRT) and the amplitude growth curve slope.^8^, ^9^, ^10^, ^11^, ^12^

Given the recent and significant technological developments in cochlear implant manufacturing, and continuous improvements in audiological diagnostic techniques, the indication criteria for cochlear implant have been extended to younger children. In the literature children with implants at age four months have already been reported.^13^

In children with implants placed before age 3 years, ECAP features essential for programming the speech processor, especially during the first year using the device. There have been few published studies on the features of ECAP recordings in children, particularly the duration of use of cochlear implants.

The purpose of this study was to assess the ECAP by using Neural Response Telemetry in children with implants placed by age 3 years. The following points were analyzed along the first year of use of cochlear implants:

1. The ECAP N1 peak amplitude and latency;
2. The slope;
3. The ECAP extrapolated threshold (p-NRT);
4. The recovery time of the auditory nerve.

## MATERIAL AND METHOD

This study was conducted in two cochlear implant programs in the state of Sao Paulo, Brazil. The Research Ethics Committees of both institutions approved the study (protocol numbers 039/2004 and 051/2006).

### Series

Participants were selected according to the following criteria:

The electronic device: a Nucleus 24 Cochlear Implant, which contains an auditory nerve compound action potential measuring system.

The age at surgery: given the importance of NRT in mapping children with implants placed before age 3 years, and the paucity of longitudinal studies in this population, subjects in this study were children with implants placed up to age 3 years.

The duration of use of the device: from the first return visit after activating the electrodes.

Table 1 shows the demographic data.

**Table 1:** Demographic data of subjects in this study.

Subject	Initials	Age**	Etiology	Cochlear implant	Return visits ***		
					1°.	2°.	3°.
S1	JHLA	1, 4	Idiopathic.	N24 R(CS)	2 months	4 months	6 months
S2	LBV	1, 4	Multifactorial*.	N24 R(CS)	2 months	5 months	9 months
S3	IS	1, 4	Idiopathic.	N24 R(ST)	3 months	6 months	9 months
S4	BSM	1,5	Idiopathic.	N24 R(CS)	2 months	5 months	8 months
S5	TL	1,6	Multifactorial*.	N24 R(CS)	2 months	7 months	10 months
S6	PGR	1,7	Multifactorial*.	N24 R(CS)	2 months	5 months	9 months
S7	FAB	1,8	Idiopathic. Gestational diabetes*.	N24 R(ST)	3 months	6 months	9 months
S8	AC	1, 10	Idiopathic.	N24 R(ST)	3 months	6 months	9 months
S9	JC	1, 10	Neuropathy	N24 R(ST)	3 months	6 months	11 months
S10	FP	2,1	Multifactorial*.	N24 R(CS)	2 months	5 months	8 months
S11	JVBF	2, 2	Genetic.	N24 R(CS)	3 months	5 months	9 months
S12	IAS	2,5	Idiopathic. Cold and fever at 5 months*.	N24 R(ST)	3 months	6 months	9 months
S13	NM	2, 6	Idiopathic.	N24 R(CS)	3 months	5 months	8 months

* Multifactorial etiology: intercurrences during delivery (prematurity, jaundice, incubator for more than 20 days, low birth weight, bronchopneumonia).

** Age at surgery: years, month.

*** Duration of use of the cochlear implant.

### Equipment

External equipment needed for the ECAP assessment protocol consisted of a Sprint speech processor, an external antenna with a 2x magnet, a connecting cable between the speech processor and the external antenna, the programming interface (PPS or CPS), and a computer for sending and receiving the neural information that was analyzed in this study.

The NRT software, version 3.1, was used for ECAP recordings; it is able to control stimulus and ECAP recording parameters.

### Procedures

The procedures in this study were the Impedance Telemetry and the Neural Response Telemetry.

Impedance Telemetry was done first to assess electrode integrity and function. Only software standardized normal impedance electrodes were used.

Each subject was assessed three times; neural responses were recorded, using NRT, on electrodes E5, E10, E15 and E20. In each electrode, the N1 peak amplitude and latency, the slope, the threshold and the recovery period were compared among the return visits. Electrodes E5 and E10 were placed in the basal portion of the cochlea, and were thus named basal electrodes; the electrodes E15 and E20 were named apical electrodes.

A speech therapist with experience in the NRT software selected, among the available features, a combination of stimulation and recording parameters to gather a valid auditory nerve response, according to the protocol described by Abbas et al.^1^ A valid neural response in this study was that with a visible N1 peak with a reproducible tracing, absence of artifacts and no amplifier saturation.^14^

### Statistical analysis

The following statistical tests were applied for data analysis: Wilcoxon’s non-parametric test, Friedman’s test, and the Equality of Proportions test. The significance level was 0.05 (5%).^15^

## RESULTS

ECAP results, a comparison among the three return visits and neural response features (the N1 peak amplitude, the latency, the amplitude growth function slope, the threshold and the recovery time) are described below.

**Chart 1:**
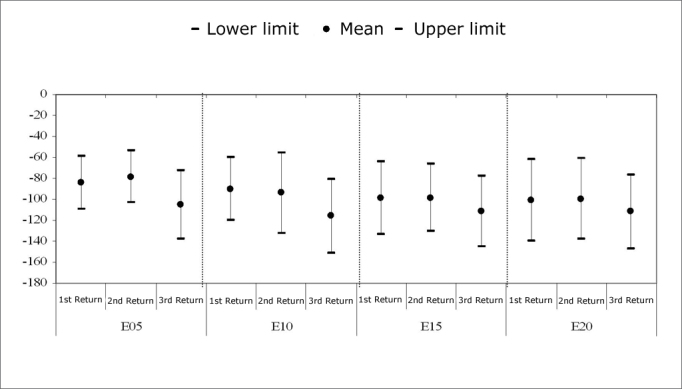
Mean and confidence interval of N1 peak amplitudes (*µ*V). Comparison among return visits. - NO KEY

Chart 1 shows the mean and confidence interval of the N1 peak (in µV), and the results of the first, second and third return visits, for each electrode.

Friedman’s test revealed a statistically significant difference among N1 peaks in each return visit for electrodes E5 (p=0.018) and E10 (p=0.023). There was an amplitude difference between the first and third return visit in electrode E5 (p=0.028), and between the second and third return visit (p=0.002). There was a significant difference only between the second and third return visit in E10 (p=0.007).

**Chart 2:**
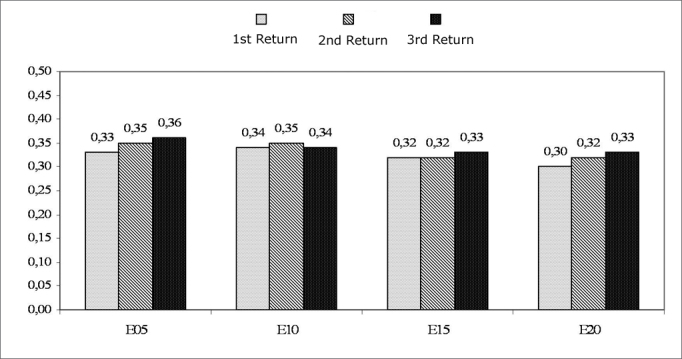
Mean of N1 peak latencies (*µ*s). Comparison among return visits. - NO KEY

Chart 2 shows the mean latency values (µs), comparing the return visit results for each electrode. Friedman’s test revealed no statistically significant differences in N1 latencies among the return visits for the electrodes E5 (p=0.101), E10 (p=0.746), E15 (p=0.751), and E20 (p=0.101).

**Chart 3:**
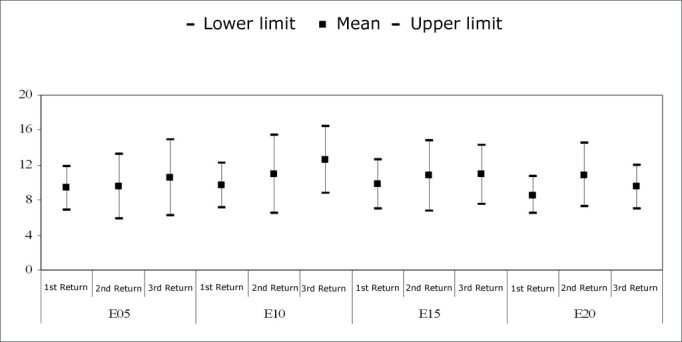
Mean and confidence intervals of slopes (*µ*V/up). Comparison among return visits. - NO KEY

Chart 3 compares the mean and confidence intervals of slope values among return visits. Friedman’s test revealed no statistically significant differences in the slopes among the return visits for the electrodes E5, E10, E15 and E20 (p=0.430, p=0.116, p=0.584, and p=0.368).

**Chart 4:**
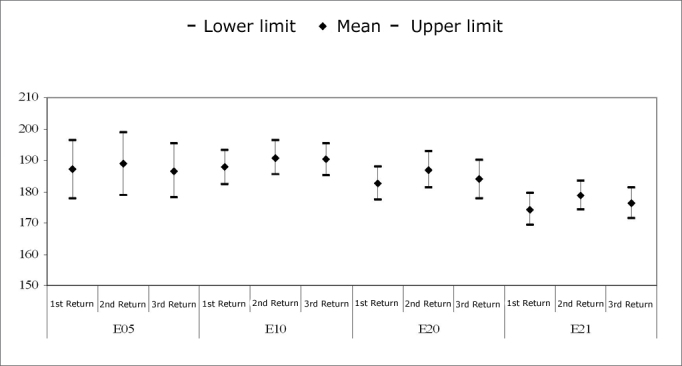
Mean and confidence interval of p-NRT (up). Comparison among return visits - NO KEY

Chart 4 compares the mean and confidence intervals of the p-NRT (up), among return visits. Friedman’s test revealed no statistically significant differences in p-NRT among return visits for electrodes E5, E10, E15, and E20 (p=0.484, p=0.584, p=0.584, and p=0.199).

**Chart 5.1:**
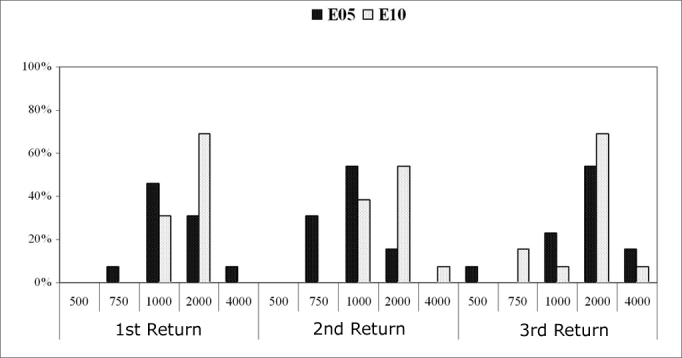
Recovery time (MPI=*µ*s) among subjects (%). Comparison among return visits for the E5 and E10 electrodes. - NO KEY

**Chart 5.2:**
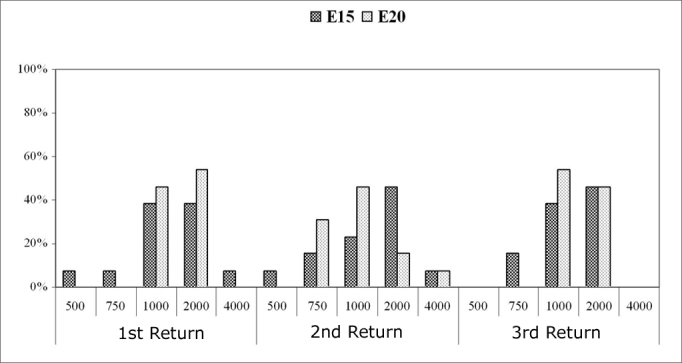
Recovery time (MPI=*µ*s) among subjects (%). Comparison among return visits for the E15 and E20 electrodes. - NO KEY

Chart 5.1 shows the recovery time for the basal electrodes E5 and E10; Chart 5.2 shows the same for the apical electrodes E15 and E20.

In electrode E5, the recovery time for most subjects (53.8%) in the first and second return visits was 1000µs. The recovery time in the third return visit for 53.8% of subjects increased to 2000µs.

In electrode E10, the recovery time for most subjects was 2000µs in the three return visits. In the first and third return visit, 69.2% of subjects had a recovery time of 2000µs; in the second return visit, this applied to 53.8% of subjects.

Recovery times measured in most subjects on electrode E15 in the first return visit were 1000µs (38.5% of subjects) and 2000µs (38.5% of subjects). The recovery time was 2000µs for 46.2% of subjects in the second and third return visits.

In electrode E20, the recovery time between the first and the second return visit decreased. The recovery time was 2000µs in the first return visit in 53.8% of subjects. This time decreased to 1000µs (46.2% of subjects) in the second return visit, and persisted in the third return visit (53.2% of subjects).

The equality of two proportions test revealed a statistically significant difference in electrode E5 between the second and the third return visit, and in electrode E20 between the first and the second return visit.

## DISCUSSION

### N1 peak amplitude

A comparison among the three return visits within the first year of cochlear implant use reveals that the N1 peak amplitude increased in all electrodes between the second and the third return visits (Chart 1). On average, the second return visit corresponded to the 5th month of cochlear implant use, and the third return visit corresponded to the 9th month of use. Amplitude differences were less evident between the first and the second return visit; the N1 peak amplitude decreased in some subjects.

The statistical analysis showed that N1 peak amplitude increases were significant in electrodes E5 and E10 (Chart 1). Amplitudes ranged from -53.73µs to -132.13µs in the second return visit, and from -72.84µs to -151.52µs, in the third return visit in the basal electrodes. No statistically significant changes were found in electrodes E15 and E20 among the return visits.

Similar results showing increased N1-P1 peak amplitudes in time have been described by other authors.^8^, ^9^, ^11^ In animal studies, Shepherd et al.^5^ found a significant amplitude increase in the EABR wave in cats during time of use of cochlear implants.

In the literature, neural response amplitude changes have been related to the long-term effects of electrical stimulation of auditory nerve fibers.

Studies of animals that were stimulated chronically revealed significantly increased neural density and preservation of the myelin sheath, which helped increase the N1 peak amplitude in segments close to the pair of stimulation electrodes.^16^, ^17^, ^18^

The results of other studies have shown that the increased neural response amplitude may be related with the fact that cochlear implant electrical stimulation changes synaptic and electrical activity on the neuronal membrane, providing neurotrophic support for auditory neurons.^19^, ^20^

Gordon et al.’s^21^ results were similar to those in our study. According to these authors, greater amplitude indicated increased synchronism among primary auditory neurons during the first year of cochlear implant use. Increased synchronism would result from the manner by which stimulation activates the primary neurons and/or from decreased firing time variations among neurons.

Other studies, however, have explained the increased neural response amplitude as being due to changes in the electrical current flow reaching neural tissues with cochlear implant use. Shepherd et al.^5^ have suggested that neural response changes in time reflect changes in the distribution of the intracochlear current, and cannot be explained by changes in the state or recruitment of auditory fibers. Other possible causes of changes in current flow reaching neural structures have been described, such as a hydric layer on the surface of electrodes, and formation of bony/fibrous tissue around the electrodes.^22^

### N1 peak latency

Our results show a lack of statistically significant differences among N1 peak latencies with time of use of cochlear implants (Chart 3), corroborating other published studies.^23^, ^24^ Dees et al.^25^ and Gordon et al.^21^, ^12^ have reported similar results. According to Haenggeli et al.^26^ and Miller et al.,^27^ direct stimulation of auditory neurons, by compensating synaptic mechanisms among cochlear hair cells and neural structures, partly explain the fact that ECAP latency is minimally affected by variations in the level of the current.

### Slope

In this study, analysis of the slope revealed no significant changes in time, as shown on Chart 3. These results are similar to other published papers in which no significant changes were found in the slope during the first year of cochlear implant use.^8^, ^10^ Although not significant, there was an increased slope value among return visits for electrodes E5, E10 and E15.

The slope, measured in µV/up, relates to auditory nerve response amplitude increases as a function of increased stimulation levels. Larger slopes are frequently described in the literature as being a result of increased neuron recruitment or increased synchronism among the same neuron population.^2^, ^14^, ^27^, ^28^, ^29^

According to Brown et al.,^30^ growth curves characterized by rapid amplitude increases as a function of the level of stimulation are reflected in the user’s ability to benefit from electrical stimulation and to process time information.

### p-NRT

The statistical analysis of the p-NRT revealed that differences among return visits were not statistically significant (Chart 4). In general, there was a slight increase in thresholds between the first and the second return visit, and a decrease of the same between the second and third return visits. On average, 1 up to 6 up variations were seen in all electrodes. The p-NRT variation was highest in the electrode E5.

Lai et al.^11^ also reported an absence of significant changes. During the first 15 months of cochlear implant use, p-NRT variations from 6 up to 11 up were described. Hughes et al.,^8^ Thai Van et al.,^9^ and Ferrari^10^ found no significant differences in the p-NRT during the first year of cochlear implant use.

Although the threshold differences were not statistically significant, this difference is considered as relevant in clinical practice. The p-NRT is the most clinically applicable measure of neural response. This value is frequently used for adjusting speech processor, especially in children with limited auditory experience.^31^, ^32^, ^25^ Thus, p-NRT variations, although not significant, may affect speech processor programming of children, at least during the first months of cochlear implant use.

### Analysis of the Recovery Time

Charts 5.1 and 5.2 show the results of auditory nerve recovery times by NRT.

Recovery time analysis showed a non-significant variation among return visits and among electrodes. The recovery time for auditory nerve fibers ranged from 1000µs to 2000µs in most subjects. The mean recovery time for electrodes E5, E10 and E15 was 2000µs along the first year of cochlear implant use. There was an improved recovery time in the E20 electrode, decreasing from 2000µs to 1000µs in most subjects.

According to Shannon,^33^ different recovery times are associated with the physiological features of each neuron, which reflects stimulation of different neural populations.

Different recovery times, ranging from 6000 to 8000µs, have been described in the literature.^34^, ^35^ These studies used surface electrodes to record neural responses in monopolar mode.

Shpak, et al.^36^ and Nelson et al.^37^ found similar results to ours. According to these authors, faster recovery times may be related to the population of children that were implanted early. In these children, a lower period of sensory deprivation may have limited the harmful effects on the status and function of auditory fibers, in particular their myelinization status.

Brown et al.’s^38^ results showed that responses recorded in electrodes E1 and E20 had faster recovery times compared to the responses in electrodes E10 and E13. According to these authors, such variability may indicate differences in stimulated neural population time processing due to myelinization, the presence of a dendritic population, and axonal integrity in the residual auditory neural population.

Brown et al.^35^ have stated that recovery time analysis is important, as it suggests an ability by auditory neurons to monitor the speech time patterns. Neuron subpopulations may limit this ability.

## CONCLUSIONS

Based on an analysis of neural responses in a sample of 13 subjects, the following conclusions in the longitudinal analysis were drawn:

- electrical stimulation by intracochlear electrodes caused no significant changes in ECAP features except for an increased N1 peak amplitude, particularly between the second and third return visits, for basal electrodes;
- the slope, N1 peak latency, and the recovery period were not different during the first year of cochlear implant use;
- the lowest amplitudes and the highest thresholds were recorded in all electrodes in the second return visit.
